# A review of the randomized clinical trial results from the *Staphylococcus aureus* network adaptive platform (SNAP) meticillin-susceptible (MSSA) and penicillin-susceptible (PSSA) domains and CloCeBa

**DOI:** 10.1093/jac/dkag215

**Published:** 2026-07-03

**Authors:** Anna L Goodman, Nicholas Easom, Dana Bielopolski, Rebecca K Sutherland, Michael Marks, Marjolein P M Hensgens, Marc Bonten, Jennifer Bostock, Genevieve Walls, David Lye, Tom Boyles, Dafna Yahav, Matthew P Cheng, Amy Legg, Mical Paul, François-Xavier Lescure, Todd C Lee, Steven Y C Tong, Joshua S Davis

**Affiliations:** Institute of Clinical Trials and Methodology, University College London Innovative Clinical Trials Unit, London, UK; Department of Infection, Guy's and St Thomas’ NHS Foundation Trust, London, UK; Department of Infection, King's College London, London, UK; Infection Research Group, Hull University Teaching Hospitals NHS Trust, Hull, UK; Department of Nephrology, Guy's and St Thomas’ Hospitals NHS Trust, London, UK; Clinical Infection Research Group, Regional Infectious Diseases Unit, Western General Hospital, Edinburgh, UK; Clinical Research Department, Faculty of Infectious and Tropical Diseases London School of Hygiene and Tropical Medicine, London, UK; Hospital for Tropical Diseases, University College London Hospitals, London, UK; Department of Infectious Diseases University Medical Center Utrecht, Utrecht, The Netherlands; Julius Center for Health Sciences and Primary Care, University Medical Center Utrecht, Utrecht University, Utrecht, The Netherlands; Julius Center for Health Sciences and Primary Care, University Medical Center Utrecht, Utrecht University, Utrecht, The Netherlands; Public Engagement Representative- no Affiliations, UK; Department of Infectious Diseases, Middlemore Hospital, Auckland, New Zealand; National Centre for Infectious Diseases, Infectious Disease Research and Training Office, Singapore; National University of Singapore, Department of Medicine, Yong Loo Lin School of Medicine, Singapore; Tan Tock Seng Hospital, Department of Infectious Diseases, Singapore; Nanyang Technological University, Lee Kong Chian School of Medicine, Singapore; Clinical Health Research Unit, Department of Medicine, University of the Witwatersrand, Johannesburg, South Africa; Infectious Diseases Unit, Sheba Medical Center, Ramat Gan Israel; Department of Medicine, Division of Infectious Diseases, McGill University, Montreal, Canada; Menzies School of Health Research, Charles Darwin University, Darwin, Northern Territory, Australia; Rambam Health Care Campus, Unit of Infectious Diseases, Haifa, Israel; The Ruth and Bruce Rappaport Faculty of Medicine, Technion Israel Institute of Technology, Haifa, Israel; Hopital Bichat - Claude-Bernard, Service des Maladies Infectieuses et Tropicales, Paris, France; Department of Medicine, Division of Infectious Diseases, McGill University, Montreal, Canada; Victorian Infectious Diseases Service, The Royal Melbourne Hospital, at the Peter Doherty Institute for Infection and Immunity, Melbourne, Australia; Department of Infectious Diseases, The University of Melbourne at the Peter Doherty Institute for Infection and Immunity, Melbourne, Australia; Melbourne Centre for Clinical Trials, School of Population and Global Health, University of Melbourne, Melbourne, Australia; Menzies School of Health Research, Charles Darwin University, Darwin, Northern Territory, Australia; Department of Infectious Diseases, John Hunter Hospital, Newcastle, NSW 2301, Australia; School of Medicine and Public Health, University of Newcastle, Newcastle, Australia

## Abstract

Current guidelines for the management of meticillin-susceptible *Staphyloccocus aureus* bloodstream infection/bacteraemia (SAB) recommend intravenous (flu)cloxacillin as the first-line treatment. This is based on decades of clinical practice. The high acute kidney injury rates seen with (flu)cloxacillin in the recently published SNAP trial, which is the largest randomized clinical trial ever in *S. aureus* bacteraemia (SAB), shows us the need to regularly test and question our usual clinical practice. Acute kidney injury is common in SAB when high doses of (flu)cloxacillin are used. The contribution of (flu)cloxacillin to developing or exacerbating acute kidney injury is likely to have been under recognized until now. Caution needs to be taken with (flu)cloxacillin. Cefazolin provides a safer and equally efficacious treatment for SAB. We discuss how this new evidence might be combined with our existing knowledge and the results of the CloCeBa trial to guide us how to appropriately manage our patients.

## Introduction

*Staphylococcus aureus* (*S. aureus*) in the bloodstream (SAB) is a life-threatening infection with an associated mortality of 15%–30% despite effective antibiotics.^1^ Mortality in *S. aureus* bacteraemia of 15% is expected in a clinical trial^2^ and reaches 30% in observational studies.^3^ These patients can be unwell at baseline, and it might be considered that these deaths are expected due to the overall frailty of the patients; however, it is estimated that 60%–75% of all deaths are infection related.^4–6^

Current guidelines for the management of meticillin-susceptible *Staphylococcus aureus* bloodstream infection/bacteraemia (SAB) recommend intravenous (flu)cloxacillin as the first-line treatment and for prolonged durations of two or more weeks.^7–9^ Although the European Society of Cardiology suggests the use of cefazolin or cloxacillin for empiric therapy in endocarditis, and cites evidence that includes both agents,^10^ their recommendation for treating *S. aureus* is oxacillin or cloxacillin.^8^ The American Heart Association guideline recommends nafcillin with a level of evidence of C (only expert opinion, case studies or standard of care).^9^ The Infectious Diseases Society of America is currently preparing the first guideline for SAB specifically. The *S. aureus* network adaptive platform (SNAP) trial has recently shown that cefazolin’s efficacy is non-inferior to (flu)cloxacillin and that (flu)cloxacillin has a higher rate of acute kidney injury (AKI) compared with cefazolin among patients with meticillin-susceptible *S. aureus* (MSSA) bacteraemia.^11^ A parallel trial within the SNAP platform exploring penicillin-susceptible *S. aureus* (PSSA) bacteraemia similarly found a higher rate of AKI using (flu)cloxacillin than the comparator (benzylpenicillin).^12^ The population of those with *S. aureus* bloodstream infection receiving antibiotics may have vulnerable kidneys due to high baseline rates of chronic kidney disease.^4^ Following publication of these SNAP trial results showing higher rates of AKI with (flu)cloxacillin, we should consider whether we are treating our patients’ kidneys safely. In the CloCeBa trial nephrotoxicity was also found to be less when cefazolin was used than when cloxacillin was used.^13^ AKI during times of acute illness increases the risk of chronic kidney disease substantially and to best manage our patients we should seek to avoid long-term kidney harm in survivors.^14^

### SNAP trial findings

The SNAP trial recently published results from the PSSA group (PSSA SNAP). In PSSA SNAP participants with PSSA bacteraemia were allocated to benzylpenicillin (referred to as 'penicillin') or (flu)cloxacillin.^15^ In the MSSA randomization (MSSA SNAP), patients with meticillin-susceptible, penicillin-resistant bacteraemia were randomized to (flu)cloxacillin or cefazolin intravenously.^15^ Separate concurrent domains are ongoing, investigating the impact of cefazolin compared to (flu)cloxacillin in children, adjunctive cefazolin in meticillin-resistant *S. aureus* (MRSA) bacteraemia, switching from IV to oral antibiotics during therapy and the role of PET/CT in SAB. A further domain exploring adjunctive clindamycin in all *S. aureus* bacteraemia has recently closed.

In the published results from PSSA SNAP AKI was observed in 17/153 (11.1%) of adult patients treated with penicillin and 27/124 (21.8%) with (flu)cloxacillin (aOR 0.50; 95% CrI 0.26–0.94; posterior probability (PP) of superiority 0.984).^12^ Baseline chronic kidney disease was 11% in the (flu)cloxacillin arm and 13% in the penicillin arm. Diabetes mellitus, a predisposing factor in renal dysfunction, was present in 34% and 31%, respectively.

In the results from MSSA SNAP, 1341 adult patients with meticillin-susceptible penicillin-resistant *S. aureus* bacteraemia were randomized, 671 to cefazolin and 670 to (flu)cloxacillin.^11^ Withdrawal or loss to follow-up was only 4% (54 patients), leaving 1287 patients in the intention-to treat analysis. The primary outcome was death at 90 days, seen in 97/645 (15%) randomized to cefazolin and 109/642 (17%) randomized to (flu)cloxacillin—adjusted odds ratio (aOR) 0.81, 95% credible interval (CI) 0.59–1.12, PP of non-inferiority 99.2% and of superiority 89.8%. SNAP uses Bayesian analysis. This result indicates a 99.2% chance that cefazolin is non-inferior to (flu)cloxacillin and 89.8% chance that it is superior for the primary outcome of 90-day all-cause mortality. In summary, cefazolin is non-inferior to (flu)cloxacillin, and has a high chance of superiority, with respect to 90 day all-cause mortality.

There were fewer serious adverse events in the cefazolin arm (aOR 0.41, 95% CrI 0.21–0.77) and fewer treatment discontinuations due to adverse events (aOR 0.21, 95% CrI 0.11–0.38). The trial was open label and there was a higher rate of changing treatment due to adverse events from (flu)cloxacillin (9.1%) than from cefazolin (1.6%) although changes in treatment due to perceived treatment failure was similar in the groups (2.4% versus 2.1%, respectively). There was less AKI within 14 days in the cefazolin arm (92/660,13.9%) than seen in the (flu)cloxacillin arm (127/648, 19.6%), (aOR 0.67; 95% CrI 0.50–0.89; probability of superiority 99.7%). There was correspondingly a reduced risk of requiring new renal replacement therapy within 90 days with cefazolin [17/668, 2.5% cefazolin versus 24/657, 4.1% (flu)cloxacillin, aOR 0.61, 95% CrI 0.33–1.11]. However, there was no statistical difference in the need for ongoing renal replacement therapy at 90 days (aOR 1.00, 95% CrI 0.31–3.16). In summary, rates of AKI were found to be higher with (flu)cloxacillin than cefazolin.

### CloCeBa trial findings

The CloCeBa trial from France reinforced the SNAP MSSA result in a smaller trial through replication of the results.^13^ With 315 patients with MSSA bacteraemia (including isolates with and without penicillin resistance) the authors also found non-inferiority of cefazolin compared to cloxacillin for the primary outcome of treatment success. This primary endpoint was met in 109 of 146 (75%) participants in the cefazolin group versus 108 of 146 (74%) participants in the cloxacillin group (treatment difference −1%; 95% CI −11 to 9; *P* = 0.012). Subsequent analysis revealed that 30% of their cases lacked the *blaZ* gene and were therefore susceptible to penicillin. AKI reporting in CloCeBa was not routine and severe adverse events relating to AKI by day 7 were found in 15/128 (12%) patients randomized to cloxacillin and 1/134 (1%) of those randomized to cefazolin [treatment difference −11% (−18 to −6), *P* = 0.0002], and by the end of study treatment 4/111(4%) and 17/99 (17%) ((treatment difference −13% (−23 to −6), *P* = 0.0008) respectively.

### Cefazolin and the treatment of infective endocarditis with SAB

The CloCeBa investigators wish to explore the theoretical cefazolin inoculum effect and will be doing further analysis on the strains to confirm the safety of cefazolin in *blaZ* positive MSSA endocarditis. In MSSA SNAP, the exclusion of PSSA enriched for isolates that had a ß-lactamase. In this 104 evaluable patients had infective endocarditis, MSSA SNAP found mortality for these patients with endocarditis was 8/51 (15.7%) for cefazolin and 13/53 (24.5%) for (flu)cloxacillin (aOR 0.54; 95%CrI 0.20–1.43; probability of non-inferiority 94.4% and superiority 88.9%).^13^

### Use of cefazolin in practice

In view of these results clinical services that are currently using (flu)cloxacillin are reviewing their practice and many are already moving to cefazolin as first-line therapy for penicillin-resistant MSSA bacteraemia. It is likely this result would be applicable to all MSSA bacteraemia, including those for which penicillin is susceptible or unknown.

In the UK the British National formulary cost (lowest NHS indicative price) of a course of cefazolin 2 g three times daily for 28 days is four times higher than the cost of flucloxacillin 2 g four times daily for 28 days. However, over a 28 day period, both figures are low in healthcare terms (£1655 versus £414, respectively) and nursing time and consumables are reduced due to cefazolin needing less request administration. In Canada cefazolin is approximately one-sixth the cost of cloxacillin, demonstrating global variation. In some areas, such as parts of Europe and the UK, cefazolin has intermittently been difficult to obtain since the incorporation of its use in surgical prophylaxis guidelines leading to a wide use of the agent. However, in South Africa, cefazolin supply is reliable but cloxacillin has frequent times of stock shortages. The authorization and addition of cefazolin to formularies of SNAP sites enabled access to this treatment throughout the trial and it is expected this will increase further with the publication of these results.

### Acute kidney injury and (flu)cloxacillin

A previous randomized clinical trial (RCT) in MRSA bloodstream infection, the CAMERA2 trial, was stopped due to safety concerns when 23% of patients receiving the addition of a ß-lactam to their usual treatment (vancomycin or daptomycin) developed AKI. This was found to be 27% (30/111) in those receiving (flu)cloxacillin (25/90, 28%) or cloxacillin (5/21, 24%) compared with 4% (1/27) in those who received adjunctive cefazolin.^16^ Need for temporary renal replacement therapy occurred more commonly in those provided with (flu)cloxacillin (4/111, 3.6%) than for vancomycin alone (1/133, 0.7%) or cefazolin (0/27, 0%).^17^ Renal replacement therapy beyond day 90 was needed in one patient provided with cloxacillin, two given cefazolin and one given both flucloxacillin and cefazolin compared with two patients on vancomycin alone.

This finding led to the subsequent selection of cefazolin rather than (flu)cloxacillin as adjunctive treatment in the ongoing MRSA trial within SNAP, but it was not anticipated that the use of (flu)cloxacillin without vancomycin for treatment of MSSA bacteraemia would again be found to be associated with such high rates of AKI.

The association between (flu)cloxacillin use and AKI has been previously established in the literature surrounding prophylaxis.^18,19^ Most studies are single centre and retrospective, and in many interpretations are complicated by use of flucloxacillin with gentamicin or comparison against vancomycin, both of which also have a degree of nephrotoxicity. Using 2 g flucloxacillin given every 6 hours, as dosed in the SNAP trial, just 24 hours of treatment for surgical prophylaxis was associated with a 8.5% rate of AKI in the largest of these studies compared with 2% with a cephalosporin 1.5–3 g cefuroxime over 24 hours.^19^ Rates were even higher at 13% when gentamicin was co-administered with flucloxacillin. In another study in Scotland, the proportion of patients with any form of AKI, defined as serum creatinine >1.5× (or fall in eGFR >25% relative to baseline), was 8%–14% with cefuroxime and 22%–52% with flucloxacillin combined with gentamicin.^18^

The mechanism by which (flu)cloxacillin may lead to AKI is speculative. In a prospective single centre pharmacokinetic pharmacodynamic (PKPD) study 50 patients with MSSA- SAB were monitored. In that study the pre-defined threshold for a potentially toxic drug concentration was an unbound trough flucloxacillin concentration of >10 times the ECOFF value (i.e. m20 mg/L). This threshold was met by nine patients and AKI was more common in that subset. However, AKI will increase the concentration of flucloxacillin and when pre-existing chronic kidney disease (which occurred in 13 of the 50 patients) was adjusted for this difference was no longer present.^20^ In the Scottish surgical prophylaxis study previously mentioned comparison was made between rates of AKI when gentamicin was given combined with either flucloxacillin at a higher-dose of 5–8 g or a lower dose of 3–4 g and were found to be 22% and 52%, respectively.^18^ Three of 198 patients needing temporary haemodialysis has all received high-dose flucloxacillin with gentamicin and biopsies in two of these three showed acute tubulo-interstitial nephritis. Renal biopsy-proven acute tubule-interstitial nephritis resulting in AKI after receiving (flu)cloxacillin has been reported.^21,22^ Whether this is related to a subclinical allergic reaction remains uncertain. Flucloxacillin is an isoxazolyl penicillin with a bulky aromatic side chain. This increases lipophilicity and intracellular accumulation in proximal renal tubular cells compared with cefazolin, which has a simpler side chain and different β-lactam nucleus, making it less prone to intracellular trapping. Cefazolin’s hydrophilic properties make it less likely to cross mitochondrial membranes to induce direct mitochondrial toxicity compared with flucloxacillin; with proximal renal tubules having the highest density of mitochondria and the main target of AKI.^23^ The observed difference in AKI between flucloxacillin and cephazolin is thus biologically plausible and may be related to its ability to induce proximal renal tubular dysfunction beyond inducing allergic interstitial nephritis.

Severe adverse events (SAE) and severe adverse reactions (SAR) are required by regulators to be reported in trials but may introduce a level of inadvertent ascertainment bias in their reporting. It is likely that the reporting is less than occurs when an event is reported routinely in data capture. In the SNAP trial AKI was collected routinely and the reported high frequency of the event is reflective of that (Table 1). For example, in the MSSA SNAP trial the frequency of AKI reported via an SAE was 1.8% for (flu)cloxacillin compared with 19.6% when measured based on routine data collection. Cases of reported SAR due to tubulointerstitial nephritis were infrequent with one case reported from cefazolin and two cases from (flu)cloxacillin. In the smaller number of participants in PSSA SNAP and CloCeBa there were no case reports of tubulointerstitial nephritis.

**Table 1.** AKI results in selected *S. aureus* bacteraemia treatment trials

Trial	Arm	*n*	Baseline CKD (%)	Percentage of AKI collected routinely (%)	Percentage of AKI SAE/SAR (%)	Percentage needing new renal replacement therapy (%)
MSSA SNAP^a11^	Cefazolin	660	14	14	0.3	0.6
MSSA SNAP^a11^	(Flu)cloxacillin	648	14	20	1.8	0.5
PSSA SNAP^a12^	(Benzyl)penicillin	156	13	11	1.3	0
PSSA SNAP^a12^	(Flu)cloxacillin	125	11	22	3.2	0
CloCeBa^13^	Cefazolin	134	13	0.75 (3.6)^b^	0	Not reported
CloCeBa^13^	Cloxacillin	128	12	12 (17)^b^	6.2	Not reported
ERADICATE^c24^	Whole cohort (ceftobiprole versus daptomycin)	389	20.7	2.3	1.3	0.3
CAMERA2^d16^	Vancomycin alone	145	∼22	6	0.5	0.7
CAMERA2^d16^	Vancomycin plus (flu)cloxacillin	111	∼22	27	≤7.5	≤3.7
CAMERA2^d16^	Vancomycin plus cefazolin	27	∼22	4	≤7.5	≤3.7
ARREST^e25^	Whole cohort (82% (flu)cloxacillin ± rifampicin)	758	18.2	Not collected	3.0	≤3
SAFO^26^	Cloxacillin ± fosfomycin	214	8.4	Not routinely collected	2.3	Not reported
DASH^f27^	Cefazolin ± daptomycin Cloxacillin ± daptomycin	76 28	33 overall (29 without daptomycin and 48 with daptomycin)	Not routinely collected	Not reported	Not reported

The definition of AKI varied in the trials and whether or not the cohort included those on haemodialysis at baseline (which would increase the denominator) varied as shown in the footnotes. Renal replacement therapy need was assessed at end of trial (percentage to two significant figures).

^a^As described in the SNAP protocol AKI in this trial was defined as a creatinine increase ≥26.5 µmol/L by d5 or ≥1.5×  from baseline by d14 (if still in hospital).^11^ Data were collected on any need for new RRT between entering the trial and 90 days later. Patients on haemodialysis at baseline were not recruited to the MSSA and PSSA groups as cefazolin is more practical to administer than other options for haemodialysis patients.

^b^In CloCeBa the readings at day 7 are cited with the end of study treatment percentage in brackets.

^c^ERADICATE collected creatinine results regularly as used for dosing. The table reports adverse events and includes those of criteria moderate or severe (sufficient to reduce or affect daily activities or greater)

^d^CAMERA2 used a definition of a creatinine increase ≥1.5 ×  from baseline by d7 or new need for renal replacement therapy by day 90

^e^ARREST used the Common Terminology Criteria for Adverse Events Grade 3 or 4 i.e. estimated glomerular filtration rate or creatinine clearance <30 mL/min/1.73 m^2^ or new dialysis or renal transplant indicated.

^f^DASH-Nephrotoxicity: Defined by the KDIGO AKI criteria (stage 1 or higher) with the modification that urine output is not included. This is specifically to be considered new or worsening renal function within 5 days of enrolment. Patients who are already receiving dialysis at enrolment were excluded from the analysis.

### Chronic kidney disease and (flu)cloxacillin

Chronic kidney disease is common within the population with *S. aureus* bacteraemia and the associated trials (Table 1). In a *post hoc* analysis of the CAMERA2 trial AKI was found to be unrelated to baseline renal impairment.^17^ In the SNAP MSSA trial, there was a high posterior probability (PP) of a reduced risk of requiring dialysis initiation during the study 90 days’ follow-up in participants receiving cefazolin compared with participants receiving (flu)cloxacillin (2.5% versus 3.7%) but there was no difference in the need for ongoing dialysis at 90 days (three versus four cases, aOR 1.00; 95% CrI 0.31–3.16; probability of superiority 50.3%). It is not clear whether the AKI occurring with (flu)cloxacillin will progress to chronic kidney failure but there is evidence from other conditions that even a mild AKI can be harmful long term, particularly when associated with sepsis.^28,29^ A study in France published in this journal recently showed how readers without easy access to cefazolin could consider a potential risk mitigation approach. Patients with MSSA SAB with an eGFR of <60 mL/min or any risk factor for AKI were treated with cefazolin from the start while those patients with no risk of AKI were treated with reduced dose cloxacillin (75–100 mg/kg/day), which was adjusted based on therapeutic drug monitoring.^30^ In the absence of therapeutic drug monitoring or cefazolin availability frequent review of blood tests and careful fluid balance should be considered at a minimum where (flu)cloxacillin continue to be used.

### High anion gap metabolic acidosis

High anion gap metabolic acidosis (HAGMA) is very rare but important to recognize when it occurs. This potentially fatal condition can occur through pyroglutamic acidosis when paracetamol is given to those receiving (flu)cloxacillin. As these treatments are commonly prescribed together and the condition is poorly recognized, reactions can indeed occur, and correct management may be delayed. A small review reports two fatal cases in the elderly related to *S. aureus* infections treated with flucloxacillin and paracetamol in combination.^31^ In the SNAP trial there was one case reported in the MSSA group. This suggests pyroglutamic acidosis could be more common than previously appreciated, with 1/670 of those receiving (flu)cloxacillin being diagnosed with this.

Chronic kidney disease is known to increase the risk of HAGMA as both the (flu)cloxacillin and paracetamol persist in the circulation increasing their effect on the gamma glutamyl cycle, and this is combined with a lower excretion of 5-oxoproline.^31^ Lactic acidosis is a type of HAGMA and there were a high number of people living with diabetes mellitus in the SNAP (33%), ARREST (30%) and ERADICATE (35%) trials.^2,11,24^ It is likely that many of those living with diabetes were taking metformin which is known to cause HAGMA in the context of haemodynamic compromise (as seen in *S. aureus* bacteraemia) and reduced kidney function.^32^ Metformin is renally cleared through a dual-pathway system with a high clearance (∼500 mL/min), freely filtered at the glomerulus as wells also actively secreted by the organic cation transporters of the proximal renal tubule. It is expected that any proximal renal tubular dysfunction induced by (flu)cloxacillin—even at a subclinical level—may increase risk of metformin accumulation and hence potential risk of metformin induced lactic acidosis. Additional risk factors include older age and frailty. In the ARREST trial 1 in 758 patients had renal tubular acidosis reported as a serious adverse event.^25^ In the ERADICATE trial 1 of 191 patients on ceftobiprole had reported metabolic acidosis and none of 198 on daptomycin.^24^ It is likely the haemodynamic compromise is a contributing factor as described.

### Electrolyte disturbance

(Flu)cloxacillin is well recognized to cause hypokalaemia in up to ∼40% of those receiving it in hospital. This contrasts with much lower rates of hypokalaemia with cephalosporins.^33^ (Flu)cloxacillin administered at doses ≥6 g/day delivers at least twice the quantity of non-re-absorbable anions compared with cefazolin, thereby exerting a greater potential to enhance potassium secretion in the distal nephron and thus cause hypokalaemia. The substantial sodium load administered via intravenous fluids during (flu)cloxacillin treatment increases the tubular flow rate within the cortical collecting duct, proportionally augmenting osmolar excretion, including that of potassium.^34^ It is common in clinical practice for clinicians to replace potassium without considering what the causative agent may be. When advising clinicians on the management of *S. aureus* bacteraemia it is incumbent on infection specialists to ensure the team managing the patient monitor electrolytes and manage them with alternative antibiotics if needed. Cefazolin may avoid these considerations in practice.

### Impact on microbiome and antimicrobial resistance

Cefazolin is a first-generation cephalosporin. Although biliary excretion is low, it may still induce gut dysbiosis including a reduction in alpha diversity and Firmicutes/Bacteroidetes (F/B) ratio in animals.^35^ Similarly, (flu)cloxacillin can also induce dysbiosis in humans,^36^ and whether (flu)cloxacillin is more gut microbiome friendly than cephazolin remains scientifically unproven. No studies to date have shown that cefazolin leads to greater antimicrobial resistance development compared with (flu)cloxacillin when used for MSSA bacteraemia. In addition, cefazolin has been demonstrated to be associated with lower collateral resistance risk compared with broader-spectrum cephalosporins.^37^ No increase in *Clostridioides difficile* infection was reported in association with cefazolin use in SNAP.

### Allergies

Approximately 10% of patients report a penicillin allergy.^38^ These patients cannot therefore receive (flu)cloxacillin without allergy assessment (although <5% have a true IgE mediated hypersensitivity). Cefazolin has a dissimilar sidechain to (flu)cloxacillin and a negligible risk of cross-reactivity (<1%).^39^ Dutch guidelines have hence advised cefazolin can be safely given to all patients, except these with severe delayed reactions, who should avoid all ß-lactam type antibiotics and be referred for specialist assessment. In those with cefazolin allergy the rate of allergy to penicillin is higher at 3.7% (95% CrI, 0.03%–13.3%).^40^ Having two ß-lactam agents available provides clear benefits in view of the superior efficacy of ß-lactams compared with glycopeptides.^41^

### Conclusion

The SNAP trial has raised our clinical level of concern regarding the use of high-dose (flu)cloxacillin and the risks of associated nephrotoxicity. Although cefazolin has a wider antimicrobial spectrum and theoretical greater risk of generating AMR, compared with (flu)cloxacillin, cefazolin has been demonstrated in a large RCT of patients with *S. aureus* bacteraemia to be non-inferior to (flu)cloxacillin and have a high probability (89.8%) of superiority for 90-day mortality and to clearly have fewer adverse effects, particularly for AKI. To minimize the renal harm to our patients we should select the best treatment, and we are seeing the rapid adoption of cefazolin as a new first-line treatment in *S. aureus* bloodstream infection.

Summary box

- Cefazolin is non-inferior and probably superior to (flu)cloxacillin for 90-day mortality in MSSA bacteraemia. Benzylpenicillin is likely to be non-inferior to (flu)cloxacillin for 90-day mortality in PSSA bacteraemia.
- Cefazolin and penicillin lead to lower AKI than (flu)cloxacillin
- Cefazolin has broader spectrum than (flu)cloxacillin (potential risk of increase in antimicrobial resistance)
- Cefazolin is safe in most of those with penicillin allergy (e.g. rash)
- Cefazolin may cause less electrolyte derangement than (flu)cloxacillin
- Paracetamol may provide additional risk of metabolic acidosis when given with (flu)cloxacillin and this is less known to occur with cefazolin
- Cefazolin is more costly in some countries but less expensive in other countries; access may be limited in certain regions

Patient box What do these results mean for patients?

We have described research in ‘*Staph. aureus* bacteraemia’ or ‘Staph blood infection’. When people have this condition, one in four people are no longer alive 3 months later.

It is very dangerous and those with kidney disease, particularly those on haemodialysis, are particularly vulnerable to the condition. The research found that our usual antibiotic for this condition, called flucloxacillin, which we have used for decades in hospitals, caused more abnormal kidney blood tests than we expected, and we saw this in at least one in five people. Using an alternative antibiotic called cefazolin it was shown that only one in seven people had that same problem. In cases where the bacteria could be treated with penicillin and was used this problem occurred in one in nine people.

Patients, carers and their families can be reassured that those caring for them can chose a safer antibiotic and patients will get the best treatment with the least side effects as standard care.

If patients get a mild rash or other side effects when taking flucloxacillin or cloxacillin they may instead be able to receive cefazolin.

To do this research, hospitals that did not previously have a treatment called cefazolin obtained it. Once they obtained it many used it routinely in those on haemodialysis as it is very practical. Instead of flucloxacillin, which is given four times a day through a vein for several weeks, cefazolin can be given after each dialysis session through the dialysis line. For this reason, patients already on dialysis were not included in the studies discussed but those with chronic kidney disease were. In one trial, called MSSA SNAP, one in seven people in with this condition had chronic kidney disease. By the end of the treatment one in 27 people who received the old treatment flucloxacillin needed new renal replacement therapy (dialysis) and fewer (one in 40) of those who received cefazolin.

## Transparency declarations

Those who received additional funding support which supported their work on these trials and to write this paper are as follows: in additional to grant support National Institute for Health and Social Care Research NIHR133719 A.L.G. was supported by the Medical Research Council (MC_UU_00004/05) in the UK. M.M. is funded by the National Institute for Health and Social Care Research (NIHR304258). The views expressed are those of the author(s) and not necessarily those of the NIHR or the Department of Health and Social Care. J.D. received funding support from the National Health and Medical Research Council and Medical Research Future Fund, Australia. G.W. was supported by the Health Research Council of New Zealand (Project grant 20/344) administered by NZ sponsor with 0.05FTE salary support; grant funding from the Athlae Lyon/Starship Foundation Grant. M.C. was supported by funding from Fonds de Recherche du Québec—Santé. S.T. was supported by funding from the National Health and Medical Research Council Australia, and Australian Medical Research Futures Fund. T.L. was supported by funding from the Canadian Institutes of Health Research; a grant from Fonds de Recherche Quebec Sante.
